# Comparative study of outcomes with total knee arthroplasty: medial pivot prosthesis vs posterior stabilized implant. Prospective randomized control

**DOI:** 10.1007/s00264-025-06420-8

**Published:** 2025-02-03

**Authors:** Bogdan Obada, Madalina Gabriela Iliescu, Dan Ovidiu Costea, Lucian Petcu, Andrei Ion Popescu

**Affiliations:** 1 Orthopaedic Traumatology Clinic, Ovidius” University of Constanta, Constanța, Romania; 2 Medical Rehabilitation Clinic, Ovidius” University of Constanta, Constanța, Romania; 3 General Surgery Clinic, Ovidius” University of Constanta, Constanța, Romania; 4 Ovidius” University of Constanta, Constanța, Romania; 5 Romanian Shoulder Institute, Ortopedicum - Orthopaedic Surgery & Sports Clinic, Bucharest, Romania

**Keywords:** Total knee arthroplasty, Medial-pivot, Posterior-stabilized, Reported outcomes, Patient satisfaction

## Abstract

**Purpose:**

Total knee arthroplasty (TKA) is an effective procedure for pain relief and restoration of function in patients with symptomatic end-stage knee arthritis. Kinematic problems due to conventional implant design have been postulated. The objective of this study is to determine if there was any difference in postoperative ROM and outcomes between patients undergoing MP-TKA vs PS-TKA.

**Methods:**

We prospectively colected the records of 600 consecutive patients with TKA performed by six senior orthopaedic surgeons between 2017 - 2021. We compared the ROM and patient-reported outcomes (Western Ontario McMaster Osteoarthritis Index WOMAC, Oxford Knee Score OKS, Knee Society Score KSS, Forgotten Joint Score FJS) between MP TKA and PS TKA.

**Results:**

There were no specific criteria for implant selection as the two groups were consecutive cohorts of patients and implant selection depended on surgeon preference. Demographics, comorbidities, diagnosis and severity of osteoarthritis were similar between MP and PS groups. The trend for OKS in our study is the same in both groups, but with higher mean values in the MP group. The trend of WOMAC pain, stiffness and disability score is the same in both groups, but with higher mean values in the PS group at one year and two years. KSS clinical and functional score is the same in both groups, but with higher mean values in the MP group. The most important score is forgetten joint score which is favourable for the MP group.

**Conclusion:**

The patients who underwent the MP-TKA scored better than those who underwent the PS-TKA, particularly regarding deep knee flexion and stability of the prosthesis. This may be related to better replication of natural knee kinematics with MP-TKA.

## Introduction

Total knee arthroplasty (TKA) is an effective procedure for pain relief and restoration of function in patients with symptomatic end-stage knee arthritis. Patient satisfaction rates have been reported between 82 and 89%. Dissatisfaction could be due to residual pain, inadequate range of motion, inability to return to normal function, abnormal gait, etc. Kinematic problems due to conventional implant design have been postulated. TKA implant designs that better recreate native knee kinematics may help to improve patient satisfaction and functional outcomes further and for that reason the knee prosthesis has undergone modifications [1–3]. To mimic normal knee kinematics, the medial stabilized prosthesis was designed. These prostheses have a highly conforming medial compartment limiting anteroposterior translation with a less conforming lateral compartment articulation allowing for an unrestricted posterolateral rollback of the lateral femoral condyle [4].

The medial pivot (MP) knee system addresses the paradoxical anterior femoral translation encountered by posterior-stabilized (PS) and it provides stability throughout the arc of motion by its inherent design physiognomies. MP TKA has been shown to be associated with greater patient satisfaction. There is limited evidence to support the use of MP design over other knee designs [5]. The MP design was invented to avoid this movement using its special asymmetric polyethylene insert, which has a highly congruent medial compartment and a less conforming lateral compartment. MP system can mimic the kinematics of the natural knee in rotation, squatting, kicking, and walking up and down the stairs. Theoretically this design avoids paradoxical anterior movement and may bring about superior patient satisfaction [6].

Posterior stabilized total knee arthroplasty (PS-TKA) consists of a femoral cam which articulates with a tibial post, improving femoral roll-back, and increasing antero-posterior and translational stability of the knee. The cam-post mechanism improves stair-climbing by the prevention of posterior tibial subluxation. The PS prosthesis substitutes the function of the PCL with the so-called cam-and-post mechanism or a scoop-shaped tibial polyethylene insert. The motion and rollback of the femoral component are mended during flexion [7]. The PS-TKA design does not restore the natural kinematics of the knee through the arc of flexion and is associated with paradoxical anterior translation of the femur with progressive knee flexion [8].

The medial knee compartment in MP-TKA function like a ball and socket joint, with the lateral femoral condyle translating in an anteroposterior direction and rotating around the medial compartment in flexion. Reported advantages of MP-TKA include improved contact stresses, reduced polyethylene wear and increased mid-flexion stability compared to PS-TKA [9, 10]. There is no uniform consensus on the impact of MP-TKA on clinical or functional outcomes compared to PS-TKA.

The objective of this study is to determine if there was any difference in postoperative ROM and clinical-outcomes between patients undergoing MP-TKA vs PS-TKA. The study hypothesis was that improved restoration of native knee kinematics with MP-TKA would improve ROM compared to PS-TKA.

## Material and methods

This study was approved by the ethical committee of Emergency Clinical County Hospital “St Apostle Andrei” of Constanta, Romania and written informed consent was obtained from all the patients. We prospectively colected the records of 600 consecutive patients with TKA performed by six senior orthopaedic surgeons between January 2017 and December 2021 in Orthopedics and Traumatology Clinic. Inclusion criteria included patients undergoing a primary TKA with a diagnosis of symptomatic osteoarthritis; patient fit for surgical intervention following review by surgeon and anaesthetist; patient able to give informed consent and agrees to comply with the postoperative review program; and patient has sufficient mobility to attend follow-up clinics. The exclusion criteria for this study included the following: patient unable to tolerate anaesthesia; patient had previous infection of knee joint; patient undergoing conversion of unicompartmental to TKA; patient has underlying neurological dysfunction compromising mobility.

Evaluation was made prospectively preoperative, postoperative at one year and at two years and there were included only the patients with complete follow-up. The data collected for analysis were age, gender, BMI, associated pathology, diagnosis, prosthesis type and postoperative complications (pain anterior knee area, biomechanical instability, clickin sound, superficial infections needed oral antibiotherapy, infection change of insert, revision for infection 2 stage surgery). There were no specific criteria for implant selection as the two groups were consecutive cohorts of patients and implant selection depended on surgeon preference.

Surgery technique was performed through a midline incision, medial parapatellar approach, subluxation and eversion of patella, intramedullar distal femur resection, extramedullary proximal tibia resection. For all the cases implant components were cemented, patella was not resurfaced, but we made patelloplasty in all cases by shaving the margins of patella to make it more conform to the implant and posterior cruciate ligament was not maintained. All operations were performed under tourniquet control. We used gap-balancing principles in both groups. Femur rotation was set to 3° and tibial slope was extablished to 3°. Local anaesthetic was injected into the surrounding tissue and posterior articular capsule after the prosthesis was placed. Tranexamic acid was injected into the articular cavity before suturing the subcutaneous tissue and a drainage tube was inserted. Anteroposterior and lateral radiographs were obtained by independent radiologists to assess the limb alignment and component positioning.

In this study, we compared the ROM and patient-reported outcomes (Western Ontario McMaster Osteoarthritis Index WOMAC, Oxford Knee Score OKS, Knee Society Score KSS, Forgotten Joint Score FJS) between the EVOLUTION MP (Microport Orthopaedics) and the Zimmer Biomet Persona PS Personalized Knee Systems [11].

All included patients were followed-up for two years following surgery. ROM was measured between maximum active extension and flexion, with patient supine by a physiotherapist using a goniometer, with no information about the prosthesis type. All study patients and observers recording clinical outcomes were blinded to the patient’s treatment group. It was not possible to blind observers recording radiological outcomes as different implant designs were used in each treatment group. To minimize bias, only the study coordinator had access to both participants’ identities and unique study numbers [12].

### Statistical analysis

The statistical analysis was performed using IBM SPSS statistics software version 25. Data is presented as mean ± standard deviation (SD) for continuous variables, or as percentages for categorical variables. An ANOVA Test with repeated measures was used to see changes to the intervention. The normality of the test variables was estimated with Kolmogorov–Smirnov Tests of Normality. Sphericity was tested with Mauchly’s test. If sphericity is violated (p < 0.05), the Greenhouse–Geisser, Huynh–Feldt and lower bound methods are used to correct the within-subjects tests. Post hoc analysis with a Bonferroni adjustment for multiple comparisons was used to discover which specific mean values differed. χ^2^ test was used to determine whether there is an association between categorical variables (i.e.,whether the variables are independent or related). The significance level α was set to 0.05.

## Results

The prospective study included 600 patients who met the inclusion criteria, divided in two groups (MP-TKA and PS-TKA) which were compared as patients-reported outcome measures after a two years follow-up. Demographics, comorbidities, diagnosis and severity of osteoarthritis were similar between MP and PS groups (Tables 1 and 2). There is no statistically significant difference between the average values ​​of age (PS 67.83 ± 6.31, MP 68.41 ± 6.65 years) and BMI (PS 28.56 ± 0.83, MP 28.45 ± 0.75) of the patients in the two groups.

**Table 1:** Comorbidities of the 2 groups

Comorbidities		Diabetes	High blood pressure	Obesity	Heart disease
PS TKA	Count (%)	85 (28.33)	229 (76.33)	48 (16)	79 (26.33)
MP TKA	Count (%)	57 (19)	190 (63.33)	33 (11)	105 (35)

**Table 2:** Diagnosis

		Primary osteoarthritis	Posttraumatic arthritis	Rheumatoid arthritis	Others	Varus	Valgus
PS TKA	Count (%)	251 (83.67)	10 (3.33)	32 (10.67)	7 (2.33)	242 (80.67)	58 (19.33)
MP TKA	Count (%)	275 (91.67)	6 (2)	14 (4.67)	5 (1.67)	267 (89)	33 (11)

Pain was major cause of patient dissatisfaction in every group. The proportion of patients presenting pain in the anterior knee area in the PS group 0.1500 (15.00%) is 2.045 times higher than the proportion of patients presenting pain in the anterior knee area in the MP group 0.0733 (7.33%); Relative Risk (RR) = 2.045, statistically significant, 95% CI for RR = (1.260, 3.319).

The proportion of patients presenting biomechanical instability in the PS group 0.1067 (10.67%) is 2.286 times higher than the proportion of patients presenting biomechanical instability in the MP group 0.0467 (4.67%); Relative Risk (RR) = 2.286, statistically significant, 95% CI for RR = (1.245, 4.195) (Table 3).

**Table 3:** Clinical evaluation

	PS Group	MP Group							
	n = 300	n = 300	χ^2^_calc_	df	p	RR	95% IC for RR	OR	95% IC for OR
Pain anterior knee area	45 (15.00%)	22 (7.33%)	8.140	1	0.004	2.045	(1.260, 3.319)	2.230	(1.303, 3.817)
Biomechanical instability	32 (10.67%)	14 (4.67%)	6.802	1	0.009	2.286	(1.245, 4.195)	2.439	(1.274, 4.671)
Clickin sound	65 (21.67%)	3 (1.00%)	61.727	1	< 0.001	21.667	(6.886, 68.174)	27.383	(8.499, 88.226)

The proportion of patients presenting clickin sound in the PS group 0.2167 (21.67%) is 21.667 times higher than the proportion of patients presenting clickin sound in the MP group 0.0100 (1.00%); Relative Risk (RR) = 21.667, statistically significant, 95% CI for RR = (6.886, 68.174).

Regarding radiographic results, none of the knees demonstrated progressive or symptomatic radiolucent lines at any point in the follow-up period. The position of all components remained unchanged, except for one knee that showed tibial component loosening in the MP group and for one knee that showed femoral component loosening in the posterior-stabilized group. No significant differences in the preoperative and postoperative patellar translation were observed between the two groups.

The proportion of patients presenting with superficial infections in the group of patients with PS (p = 0.0167) is considered equal to the proportion of patients presenting with superficial infections in the group of patients with MP (p = 0.0033): Rr = 5,000, statistically insignificant value, because the confidence interval is 95% CI = (0.588, 42.542). The proportion of patients presenting with infection—change of insert in the group of patients with PS (p = 0.0267) is considered equal to the proportion of patients presenting with infection—change of insert in the group of patients with MP (p = 0.0067): Rr = 4.000, value statistically insignificant, because the confidence interval is 95% CI = (0.856, 18.681). The proportion of patients presenting revision for infection – two stages in the group of patients with PS (p = 0.0167) is considered equal to the proportion of patients presenting revision for infection – two stages in the group of patients with MP (p = 0.0100): Rr = 1.667, statistically insignificant value, because the confidence interval is 95% CI = (0.402, 6.912). (Table 4, 5, 6, 7, 8 and 9).

**Table 4:** Infections rates of the 2 groups

	PS Group	MP Group							
	n = 300	n = 300	χ^2^_calc_	df	p	RR	95% IC for RR	OR	95% IC for OR
Superficial infections (oral AB)	5 (1.67%)	1 (0.33%)	2.694	1	0.101	5.000	(0.588, 42.542)	5.068	(0.589, 43.639)
Infection – change of insert	8 (2.67%)	2 (0.67%)	3.661	1	0.056	4.000	(0.856, 18.681)	4.082	(0. 860, 19.385)
Revision for infection – 2 stage surgery	5 (1.67%)	3 (1.00%)	0.507	1	0.477	1.667	(0.402, 6.912)	1.678	(0.397, 7.085)

**Table 5:** Functional evaluation. Patient Outcome. Range of motion

	Group		Mean	Median	SD	Min	Max	Percentiles	
Statistics		N						P25	P75
ROM preop	PS-TKA	300	94.06	94.00	1.26	90.00	96.00	93.00	95.00
	MP-TKA	300	96.60	97.00	2.97	80.00	110.00	96.00	98.00
ROM 1 year	PS-TKA	300	110.91	111.00	1.52	100.00	117.00	110.00	112.00
	MP-TKA	300	113.14	114.00	4.26	90.00	130.00	113.00	114.00
ROM 2 years	PS-TKA	300	113.64	114.00	1.74	105.00	117.00	112.00	115.00
	MP-TKA	300	114.62	115.00	4.26	93.00	135.00	114.00	115.00

**Table 6:** Functional evaluation. Patient Outcome. OKS score

	Group		Mean	Median	SD	Min	Max	Percentiles	
Statistics		N						P25	P75
OKS preop	PS-TKA	300	22.03	22.00	1.15	19.00	24.00	21.00	23.00
	MP-TKA	300	22.50	22.00	6.15	14.00	77.00	21.00	23.00
OKS 1 year	PS-TKA	300	41.07	41.00	1.28	37.00	44.00	40.00	42.00
	MP-TKA	300	41.46	42.00	1.85	33.00	46.00	41.00	42.00
OKS 2 years	PS-TKA	300	42.02	42.00	1.29	38.00	45.00	41.00	43.00
	MP-TKA	300	43.48	44.00	2.25	30.00	50.00	43.00	44.00

**Table 7:** Functional evaluation. Patient Outcome. WOMAC score

	Group		Mean	Median	SD	Min	Max	Percentiles	
Statistics		N						P25	P75
WOMAC score pain preop	PS-TKA	300	12.91	13.00	1.20	11.00	15.00	12.00	14.00
	MP-TKA	300	14.10	14.00	2.78	13.00	60.00	13.00	15.00
WOMAC score pain 1 year	PS-TKA	300	7.35	8.00	1.36	5.00	9.00	6.00	9.00
	MP = TKA	300	5.94	6.00	0.82	5.00	7.00	5.00	7.00
WOMAC score pain 2 years	PS-TKA	300	7.02	7.00	1.40	4.00	9.00	6.00	8.00
	MP-TKA	300	5.72	6.00	0.90	4.00	7.00	5.00	6.00
WOMAC score stiffness preop	PS-TKA	300	5.47	5.00	1.05	3.00	7.00	5.00	6.00
	MP-TKA	300	5.05	5.00	0.92	4.00	7.00	4.00	6.00
WOMAC score stiffness 1 year	PS-TKA	300	3.01	3.00	0.94	1.00	5.00	2.00	4.00
	MP-TKA	300	1.98	2.00	0.77	1.00	4.00	1.00	3.00
WOMAC score stiffness 2 years	PS-TKA	300	2.38	2.00	1.05	1.00	4.00	1.00	3.00
	MP-TKA	300	1.89	2.00	0.80	1.00	4.00	1.00	2.00
WOMAC score disability preop	PS-TKA	300	39.66	40.00	1.03	37.00	41.00	39.00	40.00
	MP-TKA	300	40.61	41.00	1.41	30.00	43.00	40.00	42.00
WOMAC score disability 1 year	PS-TKA	300	23.05	23.00	1.33	21.00	25.00	22.00	24.00
	MP-TKA	300	19.72	19.00	1.54	18.00	29.00	18.25	21.00
WOMAC score disability 2 years	PS-TKA	300	19.36	19.00	1.24	17.00	22.00	18.00	20.00
	MP-TKA	300	18.24	18.00	0.99	17.00	21.00	17.00	19.00
WOMAC total preop	PS-TKA	300	58.33	59.00	1.08	54.00	60.00	58.00	59.00
	MP-TKA	300	59.47	60.00	2.51	45.00	70.00	59.00	61.00
WOMAC total 1 year	PS-TKA	300	32.00	32.00	1.56	24.00	35.00	31.00	33.00
	MP-TKA	300	28.84	28.00	4.06	15.00	75.00	27.00	31.00
WOMAC total 2 years	PS-TKA	300	21.77	22.00	1.36	19.00	24.00	21.00	23.00
	MP-TKA	300	23.30	22.00	3.13	12.00	37.00	21.00	26.00

**Table 8:** Functional evaluation. Patient Outcome. KSS score

	Group		Mean	Median	SD	Min	Max	Percentiles	
Statistics		N						P25	P75
KSS clinical preop	PS-TKA	300	44.94	45.00	1.94	41.00	73.00	44.00	45.00
	MP-TKA	300	47.69	48.00	1.63	39.00	53.00	47.00	49.00
KSS clinical 1 year	PS-TKA	300	80.77	81.00	2.94	34.00	83.00	80.00	82.00
	MP-TKA	300	82.20	83.00	2.70	65.00	90.00	82.00	83.00
KSS clinical 2 years	PS-TKA	300	81.15	81.00	3.59	23.00	84.00	81.00	82.00
	MP-TKA	300	83.48	84.00	2.55	65.00	92.00	83.00	84.00
KSS functional preop	PS-TKA	300	44.24	44.00	1.15	40.00	46.00	44.00	45.00
	MP-TKA	300	48.04	48.00	3.10	40.00	79.00	47.00	49.00
KSS functional 1 year	PS-TKA	300	75.98	76.00	1.18	69.00	78.00	75.00	77.00
	MP-TKA	300	77.06	77.00	2.85	47.00	91.00	76.00	78.00
KSS functional 2 years	PS-TKA	300	73.00	73.00	1.15	70.00	76.00	72.00	74.00
	MP-TKA	300	76.95	76.00	2.72	70.00	92.00	76.00	77.00

**Table 9:** Functional evaluation. Patient Outcome. FJS score

	Group		Mean	Median	SD	Min	Max	Percentiles	
Statistics		N						P25	P75
FJS 1 year	PS-TKA	300	64.69	64.00	1.31	63.00	67.00	64.00	65.00
	MP-TKA	300	68.00	68.00	0.00	68.00	68.00	68.00	68.00
FJS 2 years	PS-TKA	300	75.04	75.00	1.34	73.00	77.00	74.00	76.00
	MP-TKA	300	80.00	80.00	0.00	80.00	80.00	80.00	80.00

**Fig. 1:**
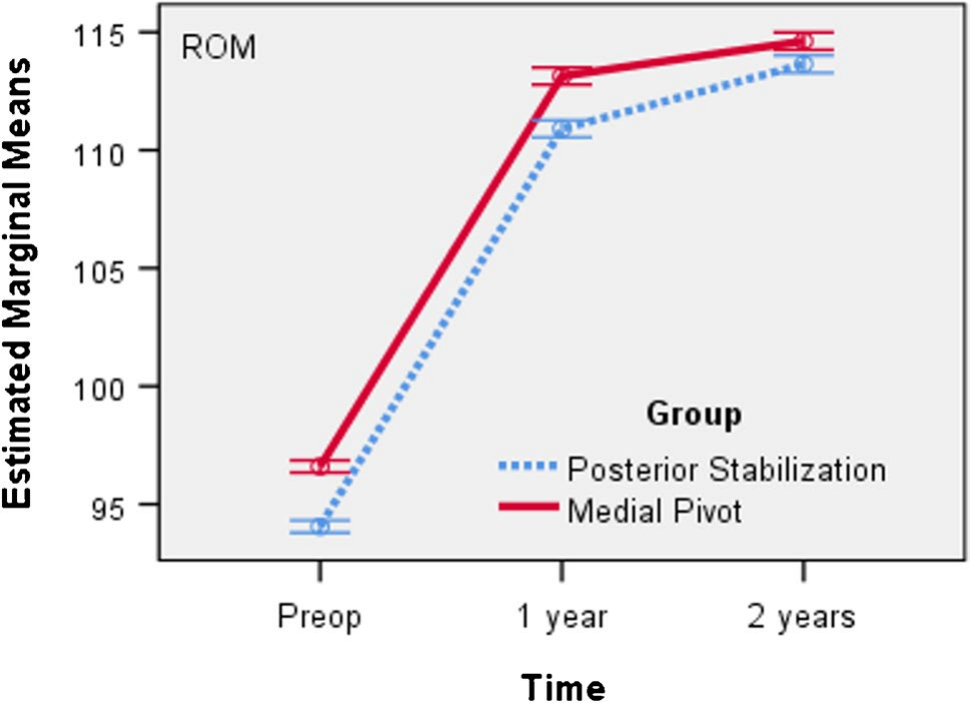
Mean ROM Score versus time

For both groups a repeated measures ANOVA with a Huynh–Feldt correction determined that mean ROM score differed statistically significantly between time points (*F*(1.708, 510.655) = 90,484.953, *p* < 0.001). We also found a statistically significant interaction effect between the grouping variable and the ROM score F (1.524, 911.286) = 44.108, p < 0.001, as suggested by the graph in Fig. 1. The trend is the same in both groups, but with higher mean values ​​in the MP group.

**Fig. 2:**
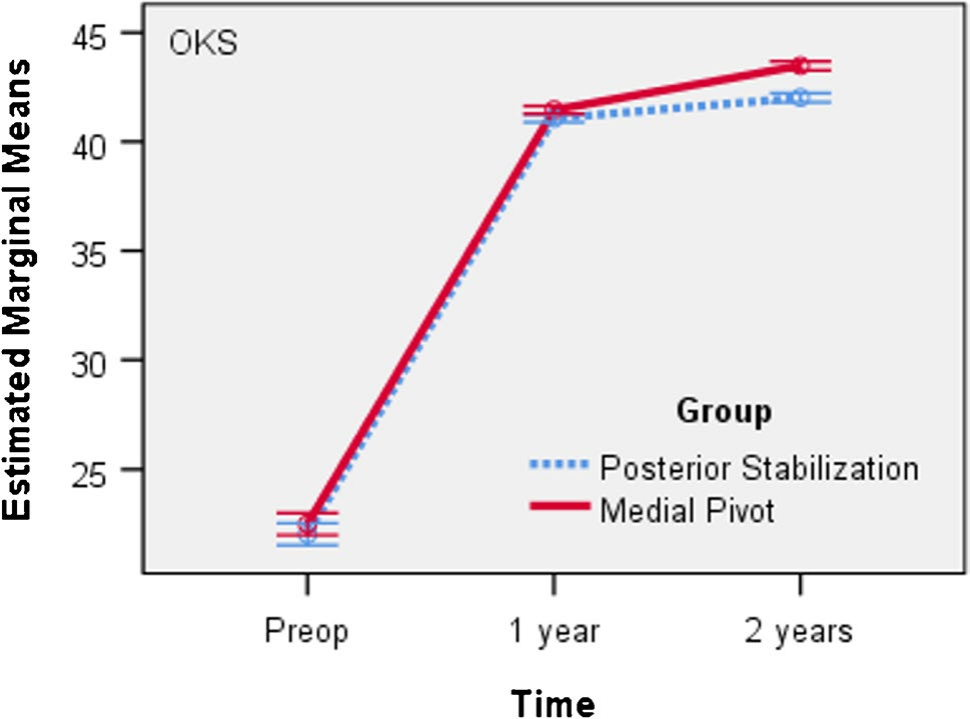
Mean OKS Score versus time

For both groups a repeated measures ANOVA with a Huynh–Feldt correction determined that mean OKS Score differed statistically significantly between time points. Post hoc analysis with a Bonferroni adjustment revealed that OKS Score was statistically significantly increased from pre-operative to one year and from one to two years. We also found a statistically significant interaction effect between the grouping variable and the OKS Score F (1.066, 638.017) = 8.728, p = 0.003, as suggested by the graph in Fig. 2. The trend is the same in both groups, but with higher mean values ​​in the MP group (Table 5).

**Fig. 3:**
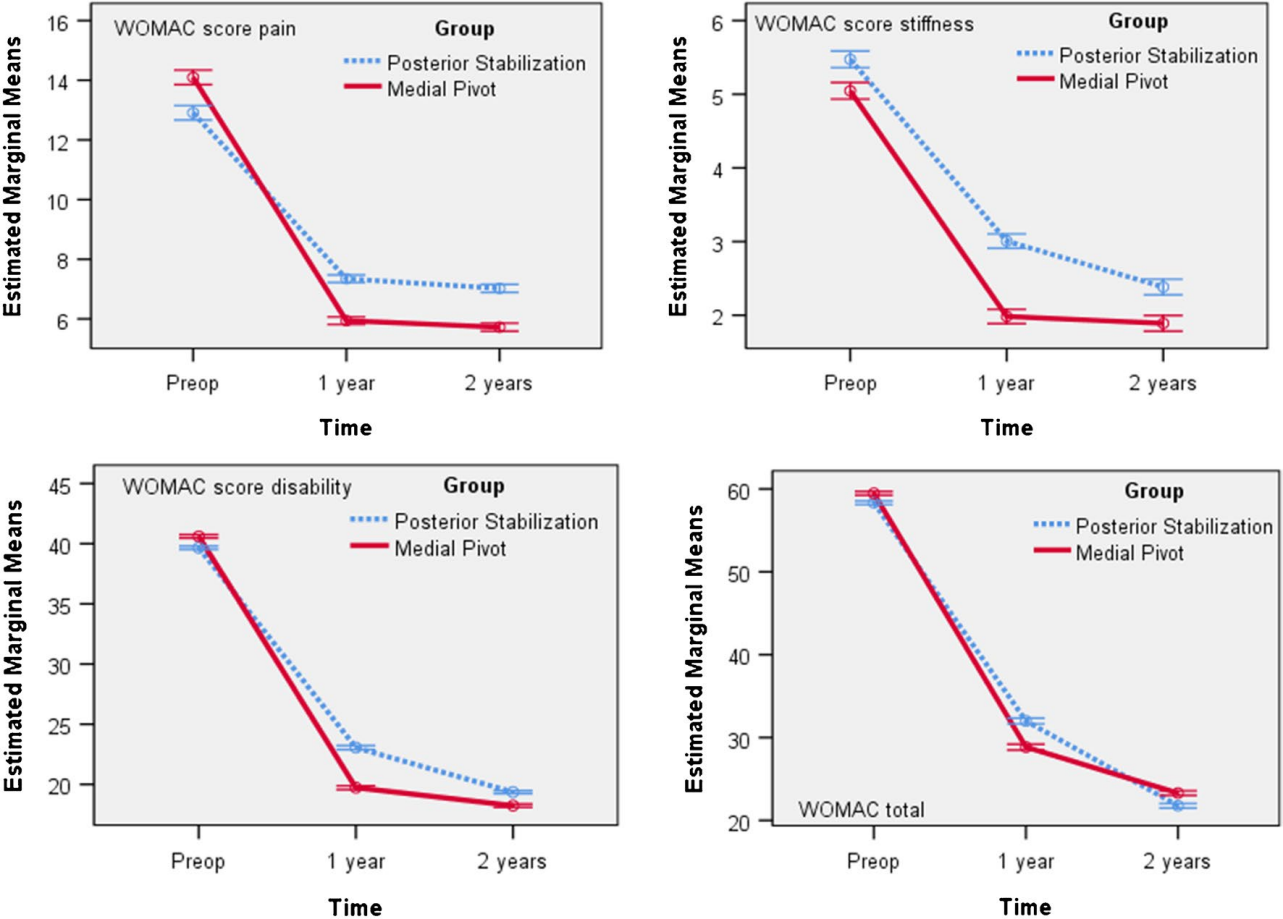
Womac mean score (pain, stiffness, disability and total)

For both groups a repeated measures ANOVA with a Greenhouse–Geisser correction determined that mean WOMAC pain, stiffness, disability and total score, differed statistically significantly between time points. Post hoc analysis with a Bonferroni adjustment revealed that WOMAC Score was statistically significantly decreased from pre-operative to one year and from one to two years. The trend of WOMAC pain, stiffness and disability score is the same in both groups, but with higher mean values ​​in the PS group at one year and two years (Fig. 3, Table 6).

**Fig. 4:**
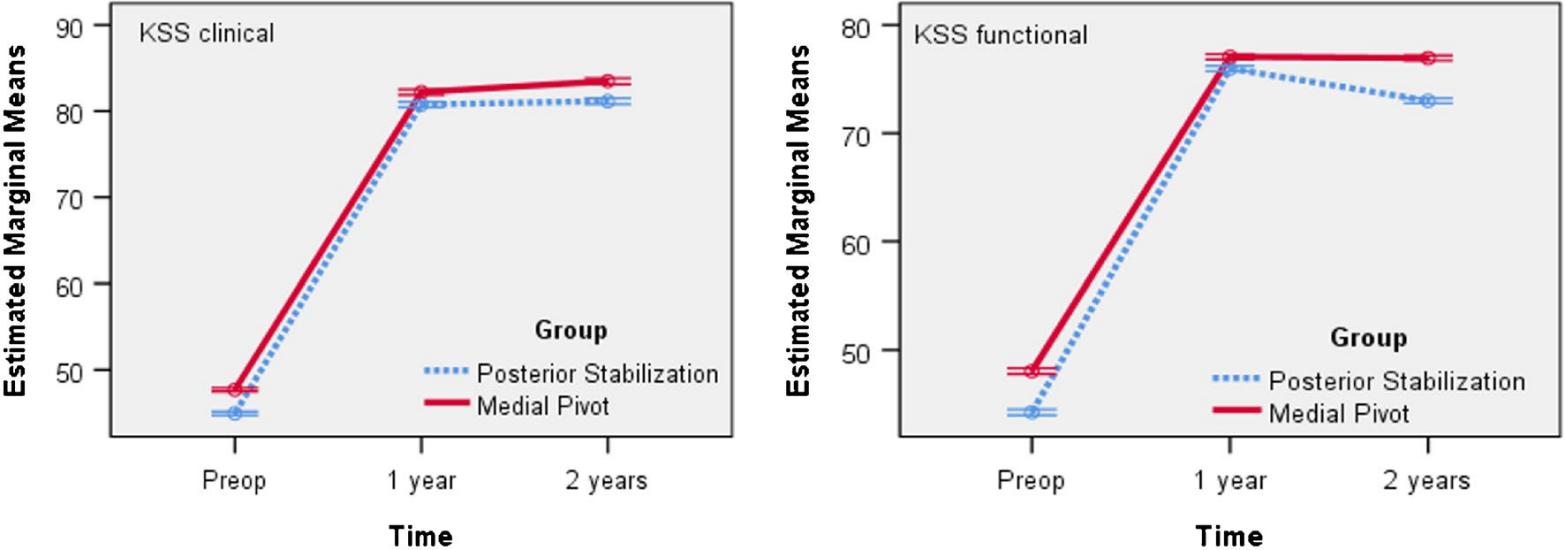
Line chart: Mean KSS clinical and functional score versus time

For both groups a repeated measures ANOVA with a Greenhouse–Geisser correction determined that mean KSS clinical and functional Score differed statistically significantly between time points (Table 7). We found a statistically significant interaction effect between the grouping variable and the KSS clinical and functional Score F (1.180, 706.157) = 21.382, p < 0.001, as suggested by the graph in Fig. 4. The trend is the same in both groups, but with higher mean values ​​in the MP group.

**Fig. 5:**
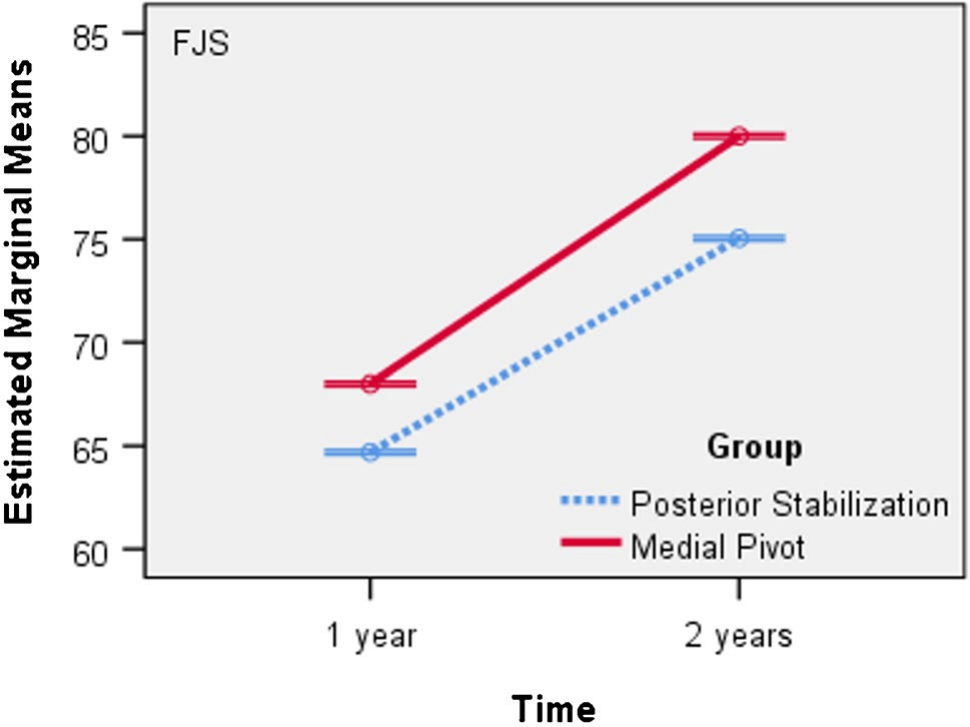
Line chart: Mean FJS Score versus time

There is a statistically significant difference in the mean FJS values between the Posterior Stabilization and Medial Pivot groups both at one year and two years (p < 0.001). Additionally, for each group, there is an increase in the mean FJS values at two years compared to 1 year (p < 0.001). Moreover, as seen in Fig. 5, the mean FJS values are lower in the PS group compared to the MP group (p < 0.001) (Table 8).

## Discussion

Patient satisfaction after TKA is one of the most important issues facing doctors and patients. Different prosthesis designs lead to varied knee kinematics and result in discrepant satisfaction rates. Despite efforts to mimic the motion of the natural knee, paradoxical anterior movement is still a common phenomenon after TKA and leads to various discomforts, such as pain caused by patellofemoral impact during flexion, which reduces patient satisfaction and joint function scores. PS prostheses may exhibit this phenomenon during normal walking, deep squatting, and other activities. MP prosthesis theoretically prevents paradoxical anterior movement [6, 9, 13].

Both PS and MP designs have shown good results in follow up studies when patients outcomes were evaluated through radiography or specific health status measures for knee OA, including American Knee Society Score, Western Ontario and McMaster Universities Osteoarthritis Index (WOMAC), Oxford Knee Score. As already stated, MP prosthesis showed knee kinematics closer to physiological condition, reduction of polyethylene wear process and better clinical outcomes, compared to PS design. Steinbrück et al. in an in-vitro study, showed reduced contact pressure on the medial compartment for the MP prosthesis when compared to the PS prosthesis at different flexion degrees. Contrasting results were found regarding knee flexion–extension range of motion (RoM), with some authors claiming a significant RoM increment for MP design compared to PS design, while other authors showed no difference between these prostheses [14, 15].

The trend for OKS in our study is the same in both groups, but with higher mean values ​​in the MP group. The trend of WOMAC pain, stiffness and disability score is the same in both groups, but with higher mean values ​​in the PS group at one year and two years. KSS clinical and functional score is the same in both groups, but with higher mean values ​​in the MP group. The most important score is forgetten joint score which is favourable for the MP group in our study.

Multiple studies have reported favourable outcomes for the MP, with satisfactory results at medium to long-term follow-up. Fan et al. reported improvements in the ROM and pain scores at five year follow-up. Bordini et al. also reported good outcomes of the MP-TKA, having a ten year survivorship estimate of 96.6%, higher than all other cemented TKA. The highly conforming design of the MP-TKA may contribute to reduced polyethylene wear and osteolysis, resulting in improved survivorship [16–18].

The MP motion is important for the prevention of patellofemoral complications because it reduces the patellofemoral contact pressure, suggesting that restoration of normal tibiofemoral kinematics may result in a decreased risk of patellofemoral problems, such as anterior knee pain. One randomized controlled trial (RCT) showed a greater ROM with a medially conforming ball-and-socket prosthesis compared with the PS-TKA at both one year and two year follow-up. The trend of ROM is the same in both groups, but with higher mean values ​​in the MP group. Contrary, another study reported a worse outcome for an MP-TKA compared with a mobile-bearing prosthesis [19].

FJS provides a better measure of high-end functionality post arthroplasty. Patients' expectations of outcomes after surgery have dramatically changed over the last years, as patients are now expecting higher levels of functionality after TKA. The FJS has been validated in several studies. Very few studies have used the FJS to compare a fixed bearing TKA with a mobile-bearing TKA. Several studies have been carried out comparing the MP-TKA with the PS-TKA using other patient-reported outcome measures. These include the WOMAC, SF-36, Knee Society Score (KSS), and Oxford Knee Score [20–22].

At present, there are a few studies on the use of the FJS, which has the advantages of a tool with high structural validity and high reliability for repeated testing, with the upper limit effect of the FJS being lower compared to WOMAC. In previous studies, patients in the MP group had a higher final FJS score than the PS group, because a high degree of stability is required when the knee joint is straightened from the flexion state. In contrast, the cam mechanism of the PS prosthesis will produce higher contact stress, which leads to knee instability, thereby affecting the ability of the knee joint to change from high flexion to straight extension. Therefore, the FJS score in the PS group was lower than that in the MP group [7, 23, 24].

Hossain et al. found a statistically significant difference in the physical elements of the SF-36 and Total Knee Function Questionnaire scoring systems, favouring the medially conforming ball-and-socket prosthesis over the PS-TKA [19]. Pritchett compared patient preference between five types of TKA design in patient with staged bilateral TKA. MP TKA was shown to have higher preference over PS TKA (76.2 vs 9.5%), cruciate retaining (CR) TKA (76 vs 12%) and mobile-bearing (MB) TKA (61.4 vs 30.1%) [25].

A retrospective study on two groups of patients by Samy reported superior FJS in MP TKA than in PS TKA (59 vs 44) at one year. The FJS in his study was somehow quite low in both groups. There were very few other studies reporting FJS as the outcome [7]. Batra et al. have found significantly better patient satisfaction and expectations in the MP group as compared to the PS group throughout the follow-up period. This study demonstrated superior sagittal knee kinematics in MP knee as compared to PS knee. The knee kinematic activity represents both the tibio-femoral and the patella-femoral joint and it involves standing from a sitting position: a routine daily activity which requires reasonable amount of mid-flexion stability. We found biomechanical instability two times more frequent in PS group than in MP group [26].

The Australian Orthopaedic Association National Joint Replacement Registry has revealed that the MP prosthesis has a higher rate of revision TKA compared with other types of prosthesis. Loosening and patellofemoral pain were the main modes of failure leading to revision, with 1.6% and 1.2% for the primary Advance MP prosthesis compared with 0.9% and 0.4% for all other types of TKA [27].

According to Papagiannis et al.’s kinematic and kinetic analysis, early outcomes could not demonstrate any notable functional and wear advantages between the two groups. Likewise, Benjamin et al.’s study revealed comparable functional grades for both PS and MP groups. However, a level I RCT by Kim et al. showed strong preferences for the non-MP implants as they obtained smaller ranges of knee motion, less patient satisfaction, and a higher complication rate in fixed bearing MP prostheses in their early outcome, despite similar long-term fixation and survival rates of both MP and PFC Sigma prostheses in their subsequent report [19, 28]. As acknowledged by Shakespeare et al. we also found most MP knees to be inherently stable, which was an objective assessment of stability by conventional methods of evaluation of achieving equal medial and lateral gaps through 0° to 90° flexion intraoperatively [29].

Nisar et al. found no significant differences in KSS or WOMAC scores when comparing MP-TKA to PS-TKA. MS TKA having no clear advantage/disadvantage in clinical or patient outcome measures, when comparing to all other implant designs, may be a result of a medial pivot motion in TKA not correlating with improved clinical outcome. Studies correlating intra-operative medial pivot patterns with post-operative outcomes have been conflicting. Nishio et al. demonstrated patients with a medial pivot pattern identified using intraoperative CT-based navigation achieved better post-operative outcomes. However, Warth et al. used intra-operative digital sensor technology to correlate intra-operative kinematic patterns with post-operative outcomes. The authors observed no difference in post-operative outcomes between those patients with a medial pivot pattern and those without [30, 31].

Macheras et al. found that the function of the knee greatly improved after TKA using MP in 325 patients, as assessed by the Western Ontario and McMaster Universities Osteoarthritis Index (WOMAC), Short Form (SF)−12, and Oxford Knee scores25. Karachalios et al. found that the 15-year cumulative survival rate of MP reached 97.7% and that the average ROM was 117 (85–130). These studies had the longest follow-up published in literature. There was also no preference in our study in prosthesis selection in relation with the deformity type, varus or valgus, similar with other findings from literature [9, 32, 33].

Persistent pain after TKA is still the major problem affecting the improvement of satisfaction in our study and the number of patients with pain in anterior knee area was double in the PS group comparative with MP group. We found a significant correlation between pain or ROM and patient satisfaction. Pain in the anterior knee can be caused by dysfunction of the patellofemoral joint in the anterior knee region, abnormalities in the patellar trajectory, and high contact stress of the patellofemoral joint after surgery. The PS prosthesis requires an intercondylar box to accommodate the column, and when the knee joint changes from flexion to extension, the patella will touch the intercondylar box. The long-term consequences are hyperplasia of the fibrous tissue nodules, which get in contact with the intercondylar box of the PS prosthesis, and cause pain in the anterior part of the knee during the extension to 30–40°. After oral administration of nonsteroidal anti-inflammatory analgesics for two months, the symptoms gradually disappeared, without adversely affecting postoperative functional recovery [34].

The phenomenon of clicking sound after knee arthroplasty is quite common. The main reason is that small nodules of the fibrous synovial hyperplasia at the superior junction of the quadriceps tendon and patella are stuck in the intercondylar fossa during movement of the knee joint. The box restricts the upward movement of the patella. When the small nodule pops out of the intercondylar box, the patella suddenly moves upwards and produces a snapping sound. The average time for the clicking sound to occur for the first time is five to 11 months. In the MP group, three patients with postoperative clicking sound was observed, while 65 patients with clicking presented in the PS group, which occurred mainly during the process of knee joint flexion to extension.

There is no difference for infection rate between the two groups. We annalysed superficial infection which was treated with oral antibiotics, early infection which needed washing and insert exchange and late infection which imposed two stage revision surgery.

Based on the findings of the present study, the choice between MP and PS TKA is still open to surgeons’ own preference. Both designs give comparable satisfactory clinical results. The incidence of postoperative complications in general is lower than the overall level, which shows that the two types of prostheses are safe and reliable. Since patient satisfaction is also affected by many other factors apart from implant design, the relative significance and the interplay between different factors may be the direction of future research.

## Limitations

We did not analyse postoperative radiographs for implant fixation and alignment. The 24-month follow-up was relatively short; however, patients with uneventful recovery are likely to have achieved optimum performance by this duration of follow-up. We studied the difference in outcomes of two implant designs with different insert geometry and commensurate difference in femoral component anatomy, hence it is not possible to determine whether the difference in outcome was due to the single radius femur or MP insert geometry. Also, the results of a prothesis type could be affected in low proportion by the experience of the surgeon, even if the surgeries were made by experienced senior surgeons.

## Conclusions

The patients who underwent the MP-TKA scored better than those who underwent the PS-TKA, particularly regarding deep knee flexion and stability of the prosthesis. This may be related to better replication of natural knee kinematics with MP-TKA. There was no significant difference in survivorship at two year follow-up between the two groups. The MP knee offers advantages to the patients who wish to perform certain activities of daily living requiring good quadriceps strength and balance.

## Data Availability

No datasets were generated or analysed during the current study.

## References

[1] Vaishya R, Scarlat MM, Bhadani J et al (2024) Ethics in orthopaedic surgery practice: balancing patient care and technological advances. Int Orthopaedics (SICOT) 48:2769–2774. 10.1007/s00264-024-06335-w 10.1007/s00264-024-06335-w 39375247

[2] Lee QJ, Wai Yee EC, Wong YC (2020) No difference in patient preference for medial pivot versus posterior-stabilized design in staged bilateral total knee arthroplasty: a prospective study. Knee Surg Sports Traumatol Arthrosc 28:3805–3809. 10.1007/s00167-020-05867-z 31993682 10.1007/s00167-020-05867-z

[3] Nisar S, Ahmad K, Palan J et al (2002) Medial stabilised total knee arthroplasty achieves comparable clinical outcomes when compared to other TKA designs: a systematic review and meta-analysis of the current literature. Knee Surg Sports Traumatol Arthrosc 30:638–665. 10.1007/s00167-020-06358-x 10.1007/s00167-020-06358-x PMC8866298 33247352

[4] Tso R, Smith J, Doma K, Grant A, McEwen P (2021) Clinical and patient-reported outcomes of medial stabilized versus non-medial stabilized prostheses in total knee arthroplasty: a systematic review and meta-analysis. J Arthroplasty 36(2):767-776.e2. 10.1016/j.arth.2020.07.086 32978025 10.1016/j.arth.2020.07.086

[5] Kulshrestha V, Sood M, Kanade S, Kumar S, Datta B, Mittal G (2020) Early outcomes of medial pivot total knee arthroplasty compared to posterior-stabilized design: a randomized controlled trial. Clin Orthop Surg 12(2):178–186. 10.4055/cios19141 32489539 10.4055/cios19141 PMC7237261

[6] Lin Y, Chen X, Li L, Li Z, Zhang Y, Fan P (2020) Comparison of patient satisfaction between medial pivot prostheses and posterior-stabilized prostheses in total knee arthroplasty. Orthop Surg 12(3):836–842. 10.1111/os.12687 32390346 10.1111/os.12687 PMC7307254

[7] Samy DA, Wolfstadt JI, Vaidee I, Backstein DJ (2018) A retrospective comparison of a medial pivot and posterior-stabilized total knee arthroplasty with respect to patient-reported and radiographic outcomes. J Arthroplasty 33(5):1379–1383. 10.1016/j.arth.2017.11.049 29276117 10.1016/j.arth.2017.11.049

[8] Dennis DA, Komistek RD, Mahfouz MR, Haas BD, Stiehl JB (2003) Multicenter determination of in vivo kinematics after total knee arthroplasty. Clin Orthop Relat Res 416:37 10.1097/01.blo.0000092986.12414.b5 14646738

[9] Harbison G, O’Donnell E, Elorza S, Howell SM, Hull ML (2024) Retention of the posterior cruciate ligament stabilizes the medial femoral condyle during kneeling using a tibial insert with ball-in-socket medial conformity. Int Orthop 48(9):2395–2401. 10.1007/s00264-024-06251-z 38997513 10.1007/s00264-024-06251-z

[10] Hossain F, Patel S, Rhee SJ, Haddad FS (2011) Knee arthroplasty with a medially conforming ball-and-socket tibiofemoral articulation provides better function. Clin Orthop Relat Res 469:55–63 20700674 10.1007/s11999-010-1493-3 PMC3008885

[11] Behrend H, Giesinger K, Giesinger JM, Kuster MS (2012) The “forgotten joint” as the ultimate goal in joint arthroplasty: validation of a new patient-reported outcome measure. J Arthroplasty 27:430-436 e1 22000572 10.1016/j.arth.2011.06.035

[12] Shi W, Jiang Y, Wang C, Zhang H, Wang Y, Li T (2020) Comparative study on mid- and long-term clinical effects of medial pivot prosthesis and posterior-stabilized prosthesis after total knee arthroplasty. J Orthop Surg Res 15(1):421. 10.1186/s13018-020-01951-9 32943092 10.1186/s13018-020-01951-9 PMC7500020

[13] Shimmin A, Martinez-Martos S, Owens J, Iorgulescu AD, Banks S (2015) Fluoroscopic motion study confirming the stability of a medial pivot design total knee arthroplasty. Knee 22:522–526 25999125 10.1016/j.knee.2014.11.011

[14] Steinbrück A, Schröder C, Woiczinski M, Fottner A, Pinskerova V, Müller PE, Jansson V (2016) Femorotibial kinematics and load patterns after total knee arthroplasty: an in vitro comparison of posterior-stabilized versus medial-stabilized design. Clin. Biomech 33:42–48. 10.1016/j.clinbiomech.2016.02.002 10.1016/j.clinbiomech.2016.02.002 26945720

[15] Esposito F, Freddolini M, Marcucci M, Latella L, Corvi A (2020) Biomechanical analysis on total knee replacement patients during gait: Medial pivot or posterior stabilized design? Clin Biomech 78:105068. 10.1016/j.clinbiomech.2020.105068 10.1016/j.clinbiomech.2020.105068 32535478

[16] Chinzei N, Ishida K, Tsumura N, Matsumoto T, Kitagawa A, Iguchi T et al (2014) Satisfactory results at 8 years mean follow-up after ADVANCE® medial-pivot total knee arthroplasty. Knee 21:387–390 24440451 10.1016/j.knee.2013.10.005

[17] Peng Y, Ding R, Li M, Wang G, Zhong Z, Wei L, Huang C, Zhang N, Hernigou P, Wang W (2024) Preoperative evaluation of femoral and tibial sagittal alignment in robotic-assisted and conventional total knee arthroplasty and consequences for practice. Int Orthop 48(8):2047–2054. 10.1007/s00264-024-06229-x 38806820 10.1007/s00264-024-06229-x PMC11246256

[18] Bordini B, Ancarani C, Fitch DA (2026) Long-term survivorship of a medial-pivot total knee system compared with other cemented designs in an arthroplasty registry. J Orthop Surg Res 11:44 10.1186/s13018-016-0388-8 PMC4837619 27094740

[19] Kim YH, Yoon SH, Kim JS (2009) Early outcome of TKA with a medial pivot fixed bearing prosthesis is worse than with a PFC mobile-bearing prosthesis. Clin Orthop Relat Res 467:493–503 18465188 10.1007/s11999-008-0221-8 PMC2628493

[20] Thienpont E, Opsomer G, Koninckx A, Houssiau F (2014) Joint awareness in different types of knee arthroplasty evaluated with the Forgotten Joint Score. J Arthroplasty 29:48–51 23688851 10.1016/j.arth.2013.04.024

[21] Obada B, Georgeanu VA, Popescu IA, Iliescu MG, Stanciu LE, Caraban BMC (2023) Late functional and radiological outcomes in recovery of patients with staged osteosynthesis for the tibial pilon fractures. Balneo PRM Res J 14:593

[22] Thomsen MG, Latifi R, Kallemose T, Barfod KW, Husted H, Troelsen A (2016) Good validity and reliability of the forgotten joint score in evaluating the outcome of total knee arthroplasty. Acta Orthop 87:280–285 26937689 10.3109/17453674.2016.1156934 PMC4900097

[23] Fahad H, Shelain P, Shin-Jae R, Sami HF (2011) Knee arthroplasty with a medially conforming ball-and-socket tibiofemoral articulation provides better function. Clin Orthop Relat Res 469(1):55–63. 10.1007/s11999-010-1493-3 20700674 10.1007/s11999-010-1493-3 PMC3008885

[24] Watanabe T, Tomita T, Fujii M, Hashimoto J, Sugamoto K, Yoshikawa H (2005) Comparison between mobile-bearing and fixed-bearing knees in bilateral total knee replacements. Int Orthop 29:179–181 15809873 10.1007/s00264-005-0646-6 PMC3456881

[25] Pitsaer E, Chergui S, Lavoie F (2024) Long-term results of a rotationally unconstrained fixed-bearing total knee prosthesis. Int Orthop 48(4):965–970. 10.1007/s00264-024-06097-5 38308765 10.1007/s00264-024-06097-5

[26] Batra S, Malhotra R, Kumar V, Srivastava DN, Backstein D, Pandit H (2021) Superior patient satisfaction in medial pivot as compared to posterior stabilized total knee arthroplasty: a prospective randomized study. Knee Surg Sports Traumatol Arthrosc 29(11):3633–3640. 10.1007/s00167-020-06343-4 33155090 10.1007/s00167-020-06343-4

[27] Brinkman JM, Bubra PS, Walker P et al (2014) Midterm results using a medial pivot total knee replacement compared with the Australian National Joint Replacement Registry data. ANZ J Surg 84:172 24165225 10.1111/ans.12428

[28] Papagiannis GI, Roumpelakis IM, Triantafyllou AI, Makris IN, Babis GC (2016) No differences identified in transverse plane biomechanics between medial pivot and rotating platform total knee implant designs. J Arthroplasty 31(8):1814–1820 26923498 10.1016/j.arth.2016.01.050

[29] Shakespeare D, Ledger M, Kinzel V (2006) Flexion after total knee replacement: a comparison between the Medial Pivot knee and a posterior stabilised implant. Knee 13(5):371–373 16828289 10.1016/j.knee.2006.05.007

[30] Higuchi H, Hatayama K, Shimizu M et al (2009) Relationship between joint gap difference and range of motion in total knee arthroplasty: a prospective randomised study between different platforms. Int Orthopaedics (SICOT) 33:997–1000. 10.1007/s00264-009-0772-7 10.1007/s00264-009-0772-7 PMC2899008 19399499

[31] Warth LC, Ishmael MK, Deckard ER, Ziemba-Davis M, Meneghini RM (2017) Do medial pivot kinematics correlate with patient-reported outcomes after total knee arthroplasty? J Arthroplasty 32(8):2411–2416 28433427 10.1016/j.arth.2017.03.019

[32] Ekhtiari S, Worthy T, Winemaker MJ, de Beer VJ, Petruccelli DT, Khanduja V, Citak M, Puri L, Wood TJ (2024) When does patient function “Plateau” after total joint arthroplasty? A cohort study. Int Orthop. 48(9):2283–2291. 10.1007/s00264-024-06248-8 39007939 10.1007/s00264-024-06248-8

[33] Iwakiri K, Ohta Y, Minoda Y, Ueno S, Kobayashi A, Nakamura H (2024) Comparative analysis of medial pivot vs. posterior stabilized total knee arthroplasty in patients with valgus deformed osteoarthritic knees: a retrospective cohort study. Int Orthop 48:3067. 10.1007/s00264-024-06337-8 39352542 10.1007/s00264-024-06337-8

[34] Petersen W, Rembitzki IV, Brüggemann GP et al (2014) Anterior knee pain after total knee arthroplasty: a narrative review. Int Orthopaedics (SICOT) 38:319–328. 10.1007/s00264-013-2081-4 10.1007/s00264-013-2081-4 PMC3923935 24057656

